# Understanding the relationship between food environments, deprivation and childhood overweight and obesity: Evidence from a cross sectional England-wide study

**DOI:** 10.1016/j.healthplace.2014.01.007

**Published:** 2014-05

**Authors:** Andreea Cetateanu, Andy Jones

**Affiliations:** a School of Environmental Sciences, University of East Anglia, Norwich, Norfolk, NR4 7 TJ, UK; b Norwich Medical School, University of East Anglia, Norwich, Norfolk NR4 7 TJ, UK; c Centre for Diet and Physical Activity Research, Box 296, Institute of Public Health, Forvie Site, Robinson Way, Cambridge CB2 0SR, UK

**Keywords:** Children, Obesity, Food environment, Deprivation, Geographic information systems

## Abstract

Using a large cross sectional English sample, we quantified the association between weight status in children aged 4–5 and 10–11 year, characteristics of the food environment, and area deprivation. We observed a positive association between the density of unhealthy food outlets in a neighbourhood and the prevalence of overweight and obesity in children. An association in the opposite direction was observed for other types of food outlets, although after adjustment this was only statistically significant for older children. The prevalence of fast food and other unhealthy food outlets explained only a small proportion of the observed associations between weight status and socioeconomic deprivation. Children׳s weight status may be influenced by their local environment, particularly older children, but associations between obesity and deprivation do not appear strongly due to local food environment characteristics.

## Highlights

• In a large sample of children, the prevalence of elevated weight status is positively associated with the presence of unhealthy food outlets in the neighbourhood.
• The opposite is true for associations with outlets selling healthy foods.
• A greater number of unhealthy food outlets are located in more deprived areas.
• The number of unhealthy food outlets only slightly explains the previously observed association between weight status and deprivation in older children.

## 1 Introduction

There is a growing body of evidence that points towards an epidemic of obesity amongst children, particularly in highly industrialised countries (Daniels, 2006). Children are an especially important group as early-life behaviours may track into adulthood and influence weight status later in life, with approximately 70% of obese children or adolescents becoming obese adults (Reilly, 2007).Obesity in children is a particular concern as it may lead to the development of asthma, psychosocial morbidity, orthopaedic and cardiovascular problems, and diabetes in childhood as well as an increased risk of obesity persistence in adulthood (Reilly, 2007). The causes of the obesity epidemic are undoubtedly multifactorial (Finegood et al., 2010; Vandenbroeck et al., 2007). Nevertheless, much attention has recently focussed on how changes to the built environment may be drivers via their influence on physical activity and dietary behaviours (Frank et al., 2007; Gordon-Larsen et al., 2006; Singh et al., 2010).

One aspect of the environment that may be particularly important in children is the availability of outlets selling low-cost energy dense foods,which particularly appeal to the young pallet (Prentice and Jebb, 2003). Within the UK, as elsewhere, the prevalence of obesity in children is known to show a gradient with social class, with obese children being more likely to come from socioeconomically deprived populations (Conrad and Capewell, 2012; Fraser and Edwards, 2010). It is also noteworthy that there is evidence of fast food and other unhealthy food outlets being more common in deprived areas in the UK (Cummins et al., 2005; Fraser and Edwards, 2010; Macdonald et al., 2007) and abroad (Pearce et al., 2007; Utter et al., 2011). On the other hand environments that are supportive of a wider range of food choice, including healthy food as defined by dietary standards (Kelly et al., 2010), are more common in higher social-class neighbourhoods (Ball et al., 2009). These social gradients are particularly pertinent given the evidence that features of the food environment are associated with both the dietary behaviours and weight status of children (Sallis and Glanz, 2009).

Despite the presence of evidence for the importance of the food environment in children, the findings from many studies are null or equivocal (Lake et al., 2010; Pearce and Witten, 2010). While some have found associations between food outlet density and weight status in children (Fraser and Edwards, 2010), or with both diet and weight (Jennings et al., 2011), and weight and deprivation (Merchant et al., 2007), others have failed to find associations between neighbourhood food outlet density and BMI in children (Sturm and Datar, 2005), or with diet (An and Sturm, 2012). This may partly be due to methodological limitations of the previous work. A key factor is that many previous studies have relied on relatively small population samples drawn from large urban areas, limiting heterogeneity in access to different types of food outlets and statistical power to detect associations. Furthermore, much of the evidence comes from the USA, a country where contrasts in urban design and neighbourhood segregation may lead to a different importance of the food environment compared to the UK (Cummins and Macintyre, 2006). Indeed, the presence of stronger residential segregation in the US (Uslaner, 2011) suggests that the local food environment may contribute more to socioeconomic differences in health (Moore and Diez Roux, 2006).

In England the recent availability of data from the National Child Measurement Programme (NCMP) provides an opportunity to provide new information on the importance of the food environment for children׳s weight status. A recent study of NCMP data (Conrad and Capewell, 2012) showed that childhood overweight and obesity rates were strongly associated with deprivation, but did not attempt to explain the reasons why this might be so. Using the whole-England sample of the NCMP for children aged 4–5 and 10–11, the present study tests a series of hypotheses. These are, firstly, area characteristics of the food environment are associated with weight-status of children in England; secondly, the strength of association will be greater for 10–11 year old children who will have more independence in the their purchasing decisions (Bowman et al., 2004; Buijzen et al., 2010), and thirdly area characteristics of the food environment mediate the association between area deprivation and child weight-status.

## 2 Methods

### 2.1 Study population

The NCMP is an England-wide cross-sectional dataset containing measured weight status recorded at school for Reception (4–5 year old) and Year 6 (10–11 year old) children (). The data has been collected on an annual basis since 2005. It provides weight status measurements, recorded using anthropometric procedures by trained staff, for approximately one million children each year attending the majority of state schools in England (NCMP, 2011a). For the purpose of this study we used the data or children in primary and secondary state maintained schools and some independent and special schools in England during the 2007/08 (*n*=9,73,073), 2008/09 (*n*=10,03,849) and 2009/10 (*n*=10,26,366) school years.

### 2.2 Outcome, predictor and confounding variables

The variables generated for this study are described in Table 1. Aggregate area-level data from the NCMP sweeps for the years 2007–8 and 2009–10 were utilised. These two periods were combined to maximise the sample size whilst restricting the period studied such that substantial changes in the food environment were unlikely to have taken place. Two outcomes were used; the prevalence of children who were overweight or obese, and the prevalence of children who were obese for 6781 geographical areas across England known as Middle Super Output Areas (MSOAs). The MSOA is a UK Census geography designed for small-area statistical analyses (ONS, 2011) with an average population of 7500. In our sample for analysis there was an average of 192 4–5 year old and 186 10–11 year old children in each MSOA. Based on standard procedure, overweight was defined as body mass index (BMI) greater than or equal to the 85th percentile and obese as a BMI greater than or equal to the 95th centile of the UK90 BMI reference (Cole et al., 2000; Reilly, 2007).

Measures of the food environment were computed in a Geographical Information System (GIS) (ArcGIS 9.3 (ESRI Inc, Redlands, CA, USA)) using the UK Ordnance Survey Points of Interest (PoI) dataset (Ordnance Survey, 2011). The PoI contains the location of all commercial facilities across England. Although concerns have been expressed regarding the accuracy of this type of facility dataset (Powell et al., 2011) recent work to validate PoI against more detailed data provided by local government for the county of Cambridgeshire, UK, concluded that PoI provided a viable alternative to other such data sources (Burgoine and Harrison, 2013). Hence it was chosen for use here.

For the purpose of this study, we extracted information on the location of all food outlets and grouped them into three categories; ‘fast food outlets’, ‘other unhealthy outlets’ and ‘mixed food outlets’. The ‘fast food outlets’ category included the PoI categories: fast food and takeaway outlets, fast food delivery services, and fish and chip shops, whilst the ‘other unhealthy outlets’ category included newsagents, convenience and general stores, and confectioners. The ‘mixed food outlets’ contained everything else and thus included cafes, pubs, restaurants, bakeries, butchers, delicatessens, fishmongers and frozen foods, green and ‘new age food outlets’, green grocers and markets, organic, cash and carry,independent supermarkets and supermarket chains. The development of the typologies was based on the evidence on associations with diet from the literature (Moore et al., 2009; Pearce et al., 2007; Sallis and Glanz, 2009) as well as fieldwork visits made by the authors to a sample of outlets falling within each category. These visits were made to ensure the classifications were appropriate to the products being sold.

Using the GIS, a count was made of the number of outlets of each type falling within the boundaries of each MSOA plus those with which it shared a boundary and this formed the primary exposure. Neighbouring MSOAs were included as the MSOA of residence was felt to represent a too restricted measure of the food environment for children. Zenk et al. (2011) have shown that most people conduct their day-to-day activities outside their residential neighbourhood. Urban MSOAs are smaller and with a higher population density compared to rural ones, and therefore by taking these units to construct our food neighbourhoods the size of a neighbourhood is associated with population density and hence the propensity of the population to travel further for food purchase.

In order to determine a robust set of relationships between weight status and the food environments, a number of covariates are considered in statistical analyses. These included the area of the food neighbourhood in square kilometres, Income Deprivation affecting Children Index (IDACI) scores that measure the proportion of children aged under 16 living in low income households (Communities and Local Government, 2011), measures of gardens and greenspace both of which have been associated with physical activity in children (Coombes et al., 2010; Jones et al., 2009), the number of similar age children as an indicator of potential social networks (Salvy et al., 2012), population ethnicity, and various indicators of area socioeconomic status.

### 2.3 Statistical analysis

Unadjusted associations between the weight status outcomes and measures of the food environment were examined using Analysis of Variance (ANOVA) and error-bar plots so that any trends were apparent, the counts of outlets in the food environments were represented as quartiles. Stepwise linear regression models were fitted to examine the relationship between the four weight status outcomes and food outlet availability scores while controlling for various covariates. All the potential covariates in Table 1 were initially included within the regression models. Those that did not show a statistically significant associations (at least at the *p*=.05 level) with each outcome were dropped in a stepwise manner until the final models retained only statistically significant variables. To determine the effect of this adjustment on the unadjusted associations observed, the quartile based measures of food outlet availability were then added to the models, and tests for trend across quartiles were made.

In order to examine associations between food outlet availability and area deprivation the Mantel–Haenszel general linear test for trend across quartiles of deprivation was used. Next, in order to examine the role of food outlet availability as a potential mediator of the relationship between area deprivation and weight status, mediation analysis was performed using the Preacher and Hayes indirect effect method (Preacher and Hayes, 2008). From this, effect ratios were computed that represent the percentage of the total effect of the independent variable on the dependent variable that is explained by the mediator (Shrout and Bolger, 2002). All statistical analyses were performed using SPSS version 19 (IBM Corp, Armonk, NY, USA).

## 3 Results

In total 279 (4.1%) of MSOAs had missing data for Reception obese, 190 (2.8%) for Reception overweight and obese, 246 (3.6%) for Year 6 obese and 239 (3.5%) for Year 6 overweight and obese. Absence was due to data suppression associated with low numbers of children participating in the NCMP in some areas. The missing MSOAs were excluded from the corresponding analyses.

**Fig. 1:**
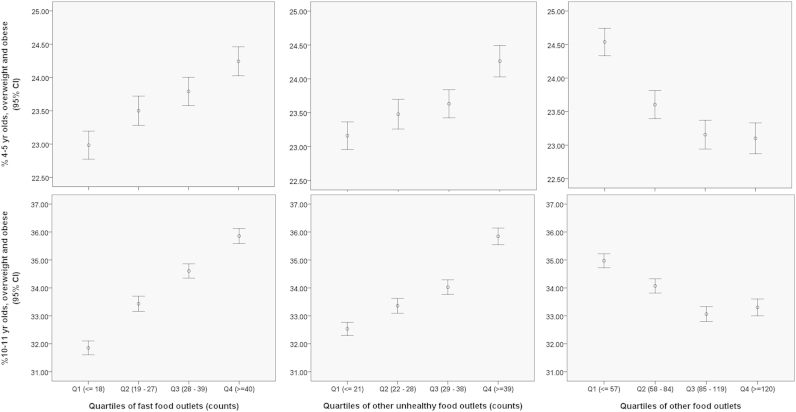


Before adjustment there was a statistically significantly (*p*<.01) increasing prevalence of overweight and obesity with a greater number of both ‘fast food’ and ‘other unhealthy’ outlets in food neighbourhoods (Fig. 1). For ‘mixed food outlets’ the direction of association was reversed. The effect size for secondary school children was greater (over 4% difference in overweight and obesity prevalence comparing the highest to lowest quartile) compared to primary school children (1.5%). Similar trends were observed for obesity alone (results not presented).

Table 2 shows the multivariable models containing the covariates that were found to be statistically significantly associated with the four outcomes. As anticipated, the prevalence of overweight and obesity was positively associated with deprivation, with a positive association with IDACI scores, and a negative association with professional employment for all outcomes. Prevalence was elevated in areas with higher non-white populations, whilst a negative association was apparent with the area of green-space and domestic gardens in each MSOA, as with the percentage of the population who were same age group peers.

Table 3 shows the associations with the four outcomes across quartiles of the food environment exposure measures after adjustment for the covariates in Table 2. For the older children there remained a statistically significant positive trend between overweight and obesity and obesity and the number of both ‘fast food’ and ‘other unhealthy’ outlets. Furthermore, there was a negative association with the availability of ‘mixed food outlets’, although the trend was somewhat attenuated from that before adjustment. For the younger children however, whilst the associations with ‘mixed food outlets’ remained unchanged as compared to the unadjusted, no association with ‘other unhealthy’ outlets remained after adjustment. For fast food outlets, a statistically significant association remained with the percentage of children who were overweight or obese, although this was in the opposite direction to that observed before adjustment, with the lowest prevalence being observed in the areas with the most outlets of this type.

Table 4 shows the unadjusted associations between the food environment measures and deprivation levels, as represented by IDACI scores. The values in the table portray, for each quartile of deprivation, the percentage of MSOAs falling within each quartile of food outlet availability. For example, 42.2% of MSOAs falling in the top quartile of fast food outlet prevalence lie in the most deprived quartile of IDACI scores, whilst just 14.1% lie in the least deprived quartile. The Mantel–Haenszel test for trend revealed a significant trend in the prevalence of all food outlets across levels of deprivation, whereby prevalence of fast food and other unhealthy food increase with area deprivation. A trend in the opposite direction was apparent for mixed food outlets.

The mediation analysis (Table 5) suggested that fast food outlet and other types of unhealthy food outlets availability partially mediated the relationship between deprivation and obesity and overweight/obesity in older children. The effect ratio is however very small, suggesting that between just 1% and 2% of the total effect of deprivation on obesity and overweight/obesity in secondary school children in England was explained by the availability of fast food and other unhealthy food outlets in the food environment. No evidence of mediation was found for mixed food outlets.

## 4 Discussion

This study found that geographical variations in measured characteristics of the food environment were associated with the prevalence of overweight and obesity in English children participating in the National Child Measurement Programme. The association was stronger for 10–11 year olds than for 4–5 year olds. There was a little evidence that food environment characteristics, mediated the known association between deprivation and weight status in this age group.

The association between deprivation and weight has been well researched, with studies consistently showing in the UK (Conrad and Capewell, 2012; Cummins et al., 2005; Kinra et al., 2000; Macdonald et al., 2007), Canada (Janssen et al., 2006), US (Singh et al., 2010), New Zealand (Pearce et al., 2007) and Europe in general (Knai et al., 2012), that overweight and obese children are more likely to come from more socio-economically deprived areas.

Another UK study also found positive associations between density of fast food outlets, deprivation and overweight and obesity, this time in children aged 3–14 years (Fraser and Edwards, 2010). A Canadian study found that children from more deprived schools have a poorer dietary intake and sit more in front of the television and computer, however there was no difference between weight status in deprived vs. the affluent schools (Merchant et al., 2007). While data on actual dietary intake was not available in our study, it was found that children from less affluent areas do have higher weight status compared to their more affluent counterparts, and there was evidence that this may be mediated by the fast food environment. It could be that the school is hence an inappropriate level to measure deprivation. One English study has reported associations between neighbourhood availability of unhealthy food outlets and weight and dietary intake in a sample of children aged 9–10 years (Jennings et al., 2011). Additionally, unhealthy food intake was associated with availability of unhealthy food outlets, which is consistent with our findings. Unlike our study which was based amongst an environmentally heterogeneous population, most studies have majorly relied on urban and relatively small population samples (Briggs and Lake, 2011; Fraser and Edwards, 2010). Where no association has been observed between food outlet density and weight status in children, this may be explained by a lack of variation in the types of environment study populations are exposed to (Sturm and Datar, 2005).

Whilst there are studies acknowledging the impact of various environment or area characteristics (such as advertisement (Buijzen et al., 2010; Story and French, 2004), family intake (Patterson et al., 1988) or deprivation (Conrad and Capewell, 2012)) on younger compared to older children, to our knowledge there are no studies assessing the impact of the food environment on children׳s weight or diet that differentiate by the age of children. Our study has shown that there seems to be different effects of the food environment characteristics, most obvious for fast food density in the neighbourhood, across children׳s age groups, with clear associations for older children, but less for younger children.

Our study has a number of strengths and limitations. The strengths of the study include the large sample size, which provides adequate statistical power. The fact that the study covered the whole population meant that there was substantial heterogeneity in both the socio-demographic characteristics of the sample as well as types of food environment to which they were exposed. The work also benefitted from the availability of an extensive number of potential confounders, and the fact that the anthropometric outcomes were measured rather than self-reported. In addition, this is one of the few studies to undertake a mediation analysis in an attempt to understand how exposure to the food environment may sit on the causal pathway between socioeconomic disadvantage and obesity. Nevertheless, there are a number of limitations to the work. The cross sectional design of the study means that caution must be taken when inferring causality of association, as with any ecological study.It is known that obese children are underrepresented in the NCMP (NCMP, 2011b) and this participation bias could reduce the heterogeneity of the outcome, thus attenuating the strength of observations. We had no information on where participants in the NCMP or their families purchased food, and hence our food neighbourhoods may not represent the locations used to actually buy food, although they do provide a measure of local purchasing potential. Indeed, childhood obesity results from an interplay of various factors which yet remain to be fully understood (Conrad and Capewell, 2012) and we did not have information on other potentially important correlates such as the physical activity levels of the children. Although continually updated, it is likely that, in common with all such products, the Points of Interest database we used may not represent all food outlets present and may contain some that have been subsequently closed. Nevertheless, recent evidence suggests that it provides an adequate representation of the food environment (Burgoine and Harrison, 2013) and it is unlikely that any omissions would have a substantial impact on the measure given the large differences in outlet density observed across the country.

We chose counts of food outlets as our outcome measure rather than density, because we were interested in looking at the number opportunities that children have, rather than how they were spatially organised. Nevertheless, to examine the impact of this decision, we performed a sensitivity analysis with counts of food outlets per unit area as the primary food exposure measures in the regression models. For fast food and other unhealthy outlets, these models were largely similar to those presented here, although a statistically significant positive association was observed between weight status and exposure to ‘mixed food outlets’ amongst Year 6 children. A comparison between the impact of different methodological choices of measuring the food environment has been described elsewhere (Burgoine et al., 2013). For each food outlet type,we also tested for the presence of the other types of food outlets in the area as potential confounders by including them as explanatory variables in the regression models, but again our results were not substantively changed and are hence not repeated here. The typology of food outlets we developed inevitably meant that difficult decisions had to be made about which category to place some food outlets. More detailed measures such as food quality ratings or store inventories might be more predictive for health outcomes, but these are costly and time consuming or do not exist on a national scale (An and Sturm, 2012).

Various methods are available for performing mediation analysis, but all have advantages and disadvantages. The classic Baron and Kenny method (Baron and Kenny, 1986) which has been used by researchers as the standard toolkit has been recently criticised (Zhao et al., 2010) and hence we chose that developed byPreacher and Hayes (2008). However, in common with other techniques, this method cannot accurately estimate the mediation effect ratio for regression models with covariates. Hence values for the indirect effects should be interpreted with caution as the method can return negative values which cannot legitimately be interpreted as a proportion; in this case, there is still mediation but the mediator acts as a suppressor variable, a situation which is referred to as inconsistent mediation (MacKinnon et al., 2007). It is also noteworthy that, whilst we found evidence of statistically significant mediation in this work, the effect ratios were small. It is likely that the level of statistical significance attained is somewhat driven by our large sample size, and therefore our findings regarding mediation should be treated accordingly.

Whilst this study supports findings in the literature that there is a direct association between area level deprivation and availability of unhealthy food, making the case for ‘food deserts’ at national level, although we recognise that evidence for their presence in the literature is equivocal (Beaulac et al., 2009; Cummins and Macintyre, 2006; Cummins et al., 2005; Pearson et al., 2005; Shaw, 2006) and most comes from the US, where there is greater neighbourhood segregation. Our findings that certain characteristics of the food environment mediated the association between deprivation and weight status in older, but not younger, children might be explained by the fact that younger children do not directly interact with their food environment as much, but they do so mostly through their parents who make choices for them, as compared with older children, who have more autonomy. Furthermore, evidence of higher provision of unhealthy food outlets in more deprived areas suggests that deprived children have more physical and economic (price of food vs. income) access to unhealthy food, a phenomenon known as the ‘obesity-hunger paradox’ or the ‘food insecurity-hunger paradox’ (Tanumihardjo et al., 2007). We believe our findings are applicable to other parts of the developed world, as the association between deprivation and obesity has also been observed in other developed countries (Knai et al., 2012; Singh et al., 2010). Studies undertaken in less developed countries report mixed associations with poverty, although it seems that by contrast, obesity in children is often a problem of the rich (Dinsa et al., 2012). How the associations we have observed may play out in such contexts is unknown.

We suggest this study highlights the importance of considering different aspects of the food environment when assessing the environmental causes of childhood obesity. Public health in the UK is changing, and some public health functions have been recently transferred from Primary Care Trusts to Local Authorities. This may present an opportunity as it will directly bring together public health practitioners and planners into the same offices for the first time. It is therefore important to better understand the association between location and health related outcomes for population health gain, as some solutions might lie in the planning domain, with fiscal and legal implications.

We suggest that public health policies to reduce obesity in children incorporate strategies to prevent high concentrations of fast food and other unhealthy food outlets. Evaluations carried out regarding zoning of food outlets around schools in New Zealand (Day and Pearce, 2011) and the US (Austin et al., 2005; Kwate and Loh, 2010; Neckerman et al., 2010) for example, found that food environments within walking proximity to schools are characterised by a high density of fast foods or other inexpensive and energy-dense food providers, and that this is particularly so in more deprived areas. Interventions for tackling childhood obesity and creating environments that are more supportive for both physical activity and better dietary choices should however nevertheless be part of the bigger picture looking at the whole obesity system, and strategies should also address the wide spectrum of factors that contribute to the obesogenic environment.

In conclusion, this study has reported evidence that, in a large and geographically diverse sample of children, whilst the density of fast food and other unhealthy food outlets in the neighbourhood may only very partially account for the observed association between childhood deprivation and childhood obesity, a higher presence of food outlets selling unhealthy food is linked to higher levels of children who are overweight and obese, while the opposite is true for food outlets selling a range of healthier food.

## Funding

AC was funded by the lord Zuckerman PhD scholarship. APJ was partially supported by the Centre for Diet and Activity Research, a UK Clinical Research Collaboration Public Health Research Centre of Excellence. Funding from the British Heart Foundation, Department of Health, Economic and Social Research Council, Medical Research Council, and the Wellcome Trust, under the auspices of the UK Clinical Research Collaboration, is gratefully acknowledged.

The funding sources had no role in the design and conduct of the study or in the collection, management, analysis, and interpretation of the data.

## Ethical approval

All data analysed was in the public domain, and no ethical approval was required.

## Figures and Tables

**Table 1:** Outcome and explanatory variables generated for Middle Super Output Areas.

**Variable description**	**Data source**	**Mean**	**SD**	**Min**	**Max**
**Outcome variables (weight status)**					
Percentage of 4–5 years old children who are overweight or obese	NCMP^a^	23.61	4.47	7.7	40
Percentage of 4–5 years old children who are obese	NCMP^a^	9.53	2.95	2.4	21
Percentage of 10–11 years old who overweight or obese	NCMP^a^	33.87	5.56	14	53.9
Percentage of 10–11 years old who are obese	NCMP^a^	18.19	4.71	4.1	36.5
**Potential covariates (neighbourhood characteristics)**					
Area square metres (adjacent MSOAs added together)	EDINA^b^	166.3	290.9	2.1	4106.8
Income deprivation affecting children (IDACI) scores, 2010	DCLG^c^	.21	.14	0	.8
Percentage area domestic gardens, 2005	ONS^d^	19.48	13.57	.1	67.9
Percentage area green space, 2005	ONS^d^	51.35	27.98	1.3	98.6
Percentage of population aged under 7 years old	Census^e^	9.68	2.03	1.9	20.6
Percentage of population aged between 10–14 years old	Census^e^	6.56	1.23	1.3	11.6
Percentage of population age 16–74 who are managers, senior officials or in a professional occupation	Census^e^	25.84	9.44	7	62.7
Percentage of population of mixed ethnicity	Census^e^	1.31	1.19	0	11.3
Percentage of population of not white or mixed ethnicity	Census^e^	7.63	13.44	0	87.1
**Primary exposure variables (food environment):**					
Counts of fast food outlets	Ordnance Survey^f^	30.38	18.06	0	266
Counts of other unhealthy food outlets	Ordnance Survey^f^	29.68	14.26	0	239
Counts of mixed food outlets	Ordnance Survey^f^	101.51	89.15	4	2255

a National Child Measurement Programme, http://www.noo.org.uk/NCMP.

b DIGIMAP http://edina.ac.uk/ukborders/.

c Department for Communities and Local Government, http://www.communities.gov.uk/communities/research/indicesdeprivation/deprivation10/.

d Office for National Statistics, http://www.neighbourhood.statistics.gov.uk/dissemination/.

e UK census of population http://casweb.mimas.ac.uk/.

f Ordnance Survey, Points of Interest, http://www.ordnancesurvey.co.uk.

**Table 2:** Associations between weight status in children and area characteristics.

	% 4–5 years old, overweight or obese				% 4–5 years old, obese				% 10–11 years old, overweight or obese				% 10–11 years old, obese			
Covariates	B	LB	UB	Sig	B	LB	UB	Sig	B	LB	UB	Sig	B	LB	UB	Sig
(Constant)	27.838	26.852	28.824	<.001	11.298	10.669	11.926	<.001	40.685	39.587	41.783	<.001	23.309	22.419	24.199	<.001
Income deprivation affecting children (IDACI) scores, 2010	9.511	8.288	10.734	<.001	7.254	6.478	8.029	<.001	10.098	8.740	11.456	<.001	11.688	10.587	12.789	<.001
Percentage area domestic gardens, 2005	−.020	−.032	−.007	.002	−.016	−.024	−.009	<.001	−.039	−.054	−.025	<.001	−.026	−.037	−.014	<.001
Percentage area green space, 2005	−.002	−.009	.005	.624	−.006	−.010	−.002	.008	−.027	−.035	−.019	<.001	−.018	−.025	−.011	<.001
Percentage of population aged under 7 years old	−.192	−.246	−.137	<.001	−.086	−.121	−.051	<.001								
Percentage of population aged 10−14 years old									−.325	−.429	−.220	<.001	−.334	−.418	−.249	<.001
Percentage of population of mixed ethnicity	.157	.032	.282	.014	.246	.167	.326	<.001	.697	.560	.833	<.001	.415	.304	.525	<.001
Percentage of population of not white or mixed ethnicity	.018	.010	.026	<.001	.017	.012	.022	<.001	.047	.039	.056	<.001	.037	.030	.044	<.001
Percentage of population age 16−74 who are managers, senior officials or in a professional occupation	−.164	−.179	−.150	<.001	−.089	−.099	−.080	<.001	−.230	−.247	−.213	<.001	−.186	−.200	−.172	<.001

*Note*: B–B slope representing the direction of effect; LB, UB – lower and upper bound of the 95% Confidence Interval (CI); and sig – significance (*p* value).

**Table 3:** Associations between weight status in children and food outlet density, after adjustment for area characteristics.

	% 4–5 years old, overweight or obese				% 4–5 years old, obese				% 10–11 years old, overweight or obese				% 10–11 years old, obese			
	B	LB	UB	Sig	B	LB	UB	Sig	B	LB	UB	Sig	B	LB	UB	Sig
Counts of fast food outlets Q2 (19–27)	.058	−.197	.313	.655	.119	−.044	.281	.153	.695	.415	.975	<.001	.479	.252	.707	<.001
Counts of fast food outlets Q3(28−39)	−.254	−.510	.002	.051	−.094	−.256	.069	.260	.880	.599	1.160	<.001	.643	.415	.871	<.001
Counts of fast food outlets Q4 (>=40)	−.597**	−.874	−.320	<.001	−.151**	−.328	.025	.093	.846**	.541	1.152	<.001	.584**	.336	.832	<.001
Counts of other unhealthy food outlets Q2 (22−28)	.016	−.240	.271	.903	.048	−.114	.211	.561	.372	.092	.653	.009	.245	.018	.473	.034
Counts of other unhealthy food outlets Q3 (29−38)	.066	−.191	.322	.617	.107	−.056	.270	.200	.628	.346	.910	<.001	.461	.232	.690	<.001
Counts of other unhealthy food outlets Q4 (>=39)	−.111	−.391	.170	.439	.018	−.161	.196	.847	.721**	.413	1.029	<.001	.511**	.262	.761	<.001
Counts of mixed food outlets Q2 (58−84)	−.275	−.534	−.017	.037	−.119	−.283	.045	.154	−.019	−.303	.266	.896	−.094	−.324	.137	.426
Counts of mixed food outlets Q3 (85−119)	−.274	−.544	−.004	.047	−.211	−.382	−.039	.016	−.281	−.579	.017	.065	−.234	−.476	.008	.058
Counts of mixed food outlets Q4 (>=120)	−.432**	−.732	−.133	.005	−.282**	−.473	−.091	.004	−.205	−.535	.126	.225	−.225	−.493	.043	.101

*Note*: Q1, Q2, Q3, Q4 represent quartiles of food outlets, with quartile 1 being the reference category in the linear regression model; B–B slope representing the direction of effect; LB, UB – lower and upper bound of the 95% Confidence Interval (CI); sig – significance (*p* value). Each set of food outlet quartiles has been introduced into the best fit model in turn.

⁎⁎ *p*<.01 represent the significance levels of the test for trend for each predictor.

**Table 4:** Unadjusted association between food environment measures and area-level deprivation.

	IDACI Q1 (<=.093) (%)	IDACI Q2 (.094–.164) (%)	IDACI Q3 (.165–.294) (%)	IDACI Q4 (.295+) (%)
Counts of fast food outlets Q1 (<=18)	35.2	32.3	19.8	12.8
Counts of fast food outlets Q2 (19–27)	29.3	25.5	26.5	18.8
Counts of fast food outlets Q3(28–39)	20.5	24.0	27.7	27.8
Counts of fast food outlets Q4 (>=40)	14.1	17.4	26.2	42.2
Counts of other unhealthy food outlets Q1 (<=20)	27.5	28.9	27.0	16.7
Counts of other unhealthy food outlets Q2 (21–27)	24.2	27.7	26.9	21.2
Counts of other unhealthy food outlets Q3 (28–36)	25.2	24.1	25.2	25.4
Counts of other unhealthy food outlets Q4 (>=37)	22.9	19.1	20.6	37.5
Counts of mixed food outlets Q1 (<=59)	17.5	21.7	31.0	29.7
Counts of mixed food outlets Q2 (60–85)	23.4	26.9	27.7	22.0
Counts of mixed food outlets Q3 (86–121)	28.0	27.5	23.8	20.6
Counts of mixed food outlets Q4 (>=122)	25.0	25.0	25.0	25.0

*Note*: the cells represent row percentages (the percentages of food outlets in each quartile across quartiles of deprivation).

**Table 5:** How neighbourhood food outlets prevalencemay mediate the association between area deprivation and child weight-status(after adjustment for area characteristics).

**Mediator**	**DV**	**IV**	**Indirect effects**	**Coefficient**	**SE**	**Bootstrapping BCa 95% CI**		**Mediation diagnosis**	**Effect ratio**
						**Lower**	**Uppe**		
Counts of fast food outlets	% 4–5 years old, overweight and obese	IDACI	Total effects	9.51	.6	–	–		
			Direct effects	9.68	.6	–	–		
			Indirect effects	−.17	.1	−.30	−.07		

Counts of fast food outlets	% 4−5 years old, obese	IDACI	Total effects	7.25	.4	−	−	Inconsistent mediation	−.01^a^
			Direct effects	7.32	.4	−	−		
			Indirect effects	−.07	0	−.15	−.02		

Counts of other unhealthy food outlets	% 4−5 years old, overweight and obese	IDACI	Total effects	9.51	.6	−	−	No	
			Direct effects	9.60	.6	−	−		
			Indirect effects	−.09	.1	−.23	.10		

Counts of other unhealthy food outlets	% 4−5 years old, obese	IDACI	Total effects	7.25	.4	−	−	No	
			Direct effects	7.26	.4	−	−		
			Indirect effects	−.01	.04	−.09	.08		

Counts of mixed food outlets	% 4−5 years old, overweight and obese	IDACI	Total effects	9.51	.6		−	No	
			Direct effects	9.58	.6	–	−		
			Indirect effects	−.07	.1	−.26	.12		

Counts of mixed food outlets	% 4–5 years old, obese	IDACI	Total effects	7.25	.4	−	−	No	
			Direct effects	7.25	.4	−	−		
			Indirect effects	.001	.1	−.12	.15		

Counts of fast food outlets	% 10–11 years old, overweight and obese	IDACI	Total effects	10.10	.7	−	−	Yes, partial	.01
			Direct effects	9.98	.7	−	−		
			Indirect effects	.12	.1	.04	.26		

Counts of fast food outlets	% 10−11 years old, obese	IDACI	Total effects	11.69	2.6	−	−	Yes, partial	.01
			Direct effects	11.60	2.6	−	−		
			Indirect effects	.08	.7	.03	.19		

Counts of other unhealthy food outlets	% 10–11 years old, overweight and obese	IDACI	Total effects	10.10	.7	–	–	Yes, partial	.02
			Direct effects	9.88	.7	–	–		
			Indirect effects	.22	.1	.10	.40		

Counts of other unhealthy food outlets	% 10–11 years old, obese	IDACI	Total effects	11.69	.6	–	–	Yes, partial	.01
			Direct effects	11.56	.6	–	–		
			Indirect effects	.13	.1	.04	.25		

Counts of mixed food outlets	% 10–11 years old, overweight and obese	IDACI	Total effects	10.10	.7	–	–	No	
			Direct effects	10.14	.7	–	–		
			Indirect effects	−.04	.1	−.23	.17		

Counts of mixed food outlets	% 10–11 years old, obese	IDACI	Total effects	11.69	.6	−	−	No	
			Direct effects	11.74	.6	−	−		
			Indirect effects	−.06	.1	−.22	.13		

*Note*: DV – dependent variable; IV – independent variable; SE – standard error; BCa – Bias Corrected and accelerated confidence interval.

a Inconsistent mediation.
